# Supplementary material for: The Wide Range of Antibiotic Resistance and Variability of Genotypic Profiles in Escherichia coli from Domestic Animals in Eastern Sicily
Source: Antibiotics (Basel). 2020 Dec 31;10(1):28. doi: 10.3390/antibiotics10010028 (PMC7823817; doi:10.3390/antibiotics10010028)
Supplement: Supplementary file 1 [file antibiotics-10-00028-s001.pdf]

antibiotics MDPI

Table S1. *E. coli* strains and antibiotic resistance profiles.

Strains	Site of isolation	Animals	Year of isolation	AMI (60 µg)*	COL (10 µg)	ENR (5 µg)	LIN/SPE (2+100 µg)	OXY (30 µg)	THP (30 µg)	TLM (15 µg)	TYL (30 µg)	TRM (1.5 µg)	SUL (25µg)	AMP (10 µg)	DOX (30 µg)	FLU (30 µg)	ERY (15 µg)	AMX (25 µg)	APR (30 µg)	Total resistance
EC1	Liver	Poultry	2018	R	S	I	I	I	R	R	R	S	R	R	S	I	R	R	R	9
EC2	Liver	Poultry	2018	R	S	S	S	I	I	S	R	I	R	S	I	S	R	R	R	6
EC3	Liver	Poultry	2018	S	S	S	R	R	R	R	R	S	R	R	R	S	R	R	S	10
EC4	Liver	Poultry	2018	R	S	S	R	R	R	R	R	S	R	R	R	S	R	R	R	12
EC5	Liver	Poultry	2018	R	S	S	I	S	I	R	R	R	R	R	I	S	R	R	R	9
EC6	Liver	Poultry	2018	R	S	R	S	R	R	R	R	R	R	R	R	R	R	R	R	14
EC7	Liver	Poultry	2018	S	S	R	S	R	R	S	R	R	R	R	R	R	R	R	R	12
EC8	Liver	Poultry	2018	S	S	R	R	R	R	S	R	R	R	R	R	R	R	R	R	13
EC9	Liver	Poultry	2018	R	S	R	S	I	I	S	R	S	R	S	I	S	I	R	S	5
EC10	Liver	Poultry	2018	R	S	R	I	R	R	R	R	R	R	R	R	R	R	R	R	14
EC11	Liver	Poultry	2018	R	R	R	S	R	R	R	R	R	R	R	R	R	R	R	R	15
EC12	Cutaneous crust	Equine	2018	S	S	S	I	R	I	S	R	R	R	R	R	S	R	R	R	9
EC13	Liver	Poultry	2018	S	S	S	S	R	R	S	R	R	R	R	R	S	R	R	S	9
EC14	Cutaneous crust	Bovine	2018	S	S	S	S	I	S	S	R	S	R	S	S	S	R	R	S	4
EC15	Mammary gland	Canine	2018	S	S	S	S	S	S	S	R	S	R	S	I	S	I	R	S	3
EC16	Liver	Bovine	2018	S	S	S	S	I	R	R	R	S	R	S	I	S	R	R	S	6
EC17	Liver	Poultry	2018	S	S	S	S	S	I	S	R	S	R	S	S	S	I	R	S	3
EC18	Liver	Poultry	2018	S	S	R	S	R	I	R	R	S	R	R	R	R	R	R	S	10
EC19	Liver	Poultry	2018	S	S	R	S	I	I	S	R	S	R	R	R	R	R	R	S	8
EC20	Joint swab	Poultry	2018	S	S	S	R	I	I	S	R	R	R	R	I	R	I	R	S	7
EC21	Liver	Poultry	2018	S	S	R	S	I	I	S	R	R	R	R	I	R	R	R	S	8
EC22	Liver	Poultry	2018	S	S	S	I	R	I	R	R	R	R	R	R	S	R	R	S	9
EC23	Liver	Poultry	2018	S	R	R	S	R	R	S	R	R	R	R	R	R	I	R	S	11
EC24	Liver	Poultry	2018	S	S	I	R	S	I	S	I	R	R	R	S	S	R	R	R	7
EC25	Liver	Poultry	2018	S	S	S	S	R	I	S	R	S	R	R	R	S	I	R	R	7
EC26	Liver	Poultry	2018	S	S	S	R	S	R	S	R	S	R	R	S	I	R	R	R	8
EC27	Liver	Poultry	2018	S	S	R	S	I	R	S	R	R	R	R	S	R	R	R	R	10

EC28	Oviduct	Poultry	2018	S	S	S	I	R	R	S	R	S	R	R	R	R	R	R	S	9
EC29	Lung	Bovine	2018	R	S	R	I	R	R	R	R	R	R	R	R	R	R	R	R	14
EC30	Liver	Bovine	2018	S	S	S	I	R	S	S	R	R	R	R	R	S	R	R	S	8
EC31	Liver	Poultry	2018	R	S	R	S	R	R	R	R	R	R	R	R	R	R	R	R	14
EC32	Kidney	Swine	2018	S	S	S	S	I	S	S	R	S	I	S	I	S	R	R	S	3
EC33	Lung	Bovine	2018	R	R	S	S	S	I	R	R	S	R	I	I	I	R	R	R	8
EC34	Liver	Poultry	2018	S	S	R	I	R	S	S	R	R	R	R	R	R	R	R	S	10
EC35	Lung	Bovine	2018	S	S	S	S	S	I	S	R	S	R	S	S	S	R	R	S	4
EC36	Lung	Rabbit	2018	R	R	R	R	R	I	I	R	R	R	R	R	R	R	R	R	14
EC37	Liver	Canary	2018	R	S	R	R	R	R	S	R	R	R	R	R	R	R	R	S	13
EC38	Brain	Swine	2018	S	S	S	S	I	S	S	R	S	R	S	I	S	R	R	S	4
EC39	Liver	Poultry	2018	S	S	S	S	R	R	S	R	S	R	R	R	S	R	R	S	8
EC40	Liver	Poultry	2018	S	S	S	S	R	R	S	R	S	R	S	R	S	R	R	S	7
EC51	Liver	Poultry	2018	S	S	S	I	R	I	S	R	S	R	S	R	S	R	R	S	6
EC52	Lung	Bovine	2018	S	S	S	I	R	S	S	R	S	R	I	R	S	R	R	R	7
EC53	Kidney	Swine	2019	R	S	S	S	S	I	R	R	S	R	R	R	S	R	R	R	9
EC54	Liver	Swine	2019	S	S	S	R	R	R	R	R	S	R	S	I	S	R	R	S	8
EC55	Liver	Poultry	2017	R	S	R	I	R	R	R	R	R	R	R	I	R	R	R	R	13
EC56	Liver	Poultry	2017	S	S	R	S	R	R	R	R	R	R	R	R	R	R	R	S	12
EC57	Liver	Poultry	2017	S	S	R	R	S	I	S	R	R	R	R	S	R	R	R	S	9
EC58	Liver	Poultry	2017	S	S	I	R	R	R	R	R	R	R	R	R	R	R	R	S	12
EC59	Lung	Bovine	2017	R	R	S	I	R	R	R	R	R	R	R	R	R	R	R	R	14
EC60	Lung	Bovine	2017	S	S	R	S	R	S	R	R	R	R	R	R	R	R	R	S	11
EC61	Liver	Poultry	2017	S	S	R	S	R	R	R	R	R	R	R	R	R	R	R	S	12
EC62	Liver	Poultry	2017	R	R	S	I	R	R	R	R	S	R	R	R	S	R	R	R	12
EC63	Liver	Poultry	2017	S	S	S	I	I	I	R	R	R	R	R	R	R	R	R	S	9
EC64	Intestine	Poultry	2017	S	S	R	R	R	R	R	R	R	R	R	R	R	R	R	S	13
EC65	Liver	Poultry	2017	S	S	R	R	R	R	R	R	R	R	R	R	R	R	R	S	13
EC66	Liver	Poultry	2017	S	S	I	S	I	R	R	R	R	R	R	I	R	R	R	S	9
EC67	Liver	Poultry	2017	R	S	I	R	R	R	R	R	R	R	R	R	R	R	R	R	14
EC68	Liver	Poultry	2017	S	S	R	S	R	R	R	R	R	R	R	R	R	R	R	S	12
EC69	Liver	Poultry	2017	S	S	I	S	R	R	R	R	R	R	R	R	R	R	R	S	11
EC70	Liver	Bovine	2017	R	S	R	R	R	R	R	R	R	R	R	S	R	R	R	S	13
EC71	Liver	Poultry	2016	S	S	R	R	R	R	R	R	R	R	R	R	R	R	R	S	13

EC72	Intestine	Poultry	2016	R	S	I	R	R	I	R	R	R	R	R	R	I	R	R	R	12
EC73	Mammary gland	Bovine	2017	S	S	S	R	I	I	R	R	S	R	S	S	S	R	R	S	6
EC74	Intestine	Swine	2016	S	S	S	R	I	I	R	R	S	R	I	S	S	R	R	S	6
EC75	Liver	Poultry	2016	S	S	S	S	R	R	R	R	R	R	R	R	S	R	R	S	10
EC76	Intestine	Swine	2016	S	S	S	R	I	R	R	R	S	R	I	S	S	R	R	S	7
EC77	Mammary gland	Bovine	2016	S	S	S	R	S	I	S	R	S	R	S	I	S	R	R	S	5
EC78	Mammary gland	Bovine	2016	S	S	S	R	R	R	R	R	S	R	R	R	S	R	R	S	10
EC79	Cerebrospinal Fluid	Bovine	2019	R	S	R	R	I	R	R	R	R	R	R	I	R	R	R	S	12
EC80	Lung	Bovine	2019	S	S	S	I	R	I	S	R	S	R	S	S	S	R	R	S	5
EC81	Liver	Bovine	2019	S	S	S	I	R	I	S	R	S	R	S	S	S	R	R	S	5
EC82	Lung	Bovine	2019	R	S	R	R	I	R	R	R	R	R	R	R	R	R	R	R	14
EC83	Lung	Bovine	2019	R	R	R	R	R	I	R	R	R	R	R	I	R	R	R	R	14
EC84	Liver	Poultry	2019	S	S	S	R	I	I	R	R	S	R	R	S	S	R	R	S	7
EC85	Liver	Poultry	2019	R	R	S	R	I	I	R	R	S	R	R	I	S	R	R	R	10
EC86	Spleen	Swine	2019	S	S	S	R	I	I	R	R	S	R	R	I	S	R	R	S	7
EC87	Liver	Poultry	2016	S	S	S	R	R	R	R	R	S	R	S	R	S	R	R	S	9
EC88	Liver	Poultry	2016	I	S	I	S	R	I	R	R	R	R	R	R	I	R	R	R	10
EC89	Mammary gland	Bovine	2015	R	S	S	S	R	I	S	R	R	R	R	R	S	R	R	S	9
EC90	Liver	Poultry	2015	S	S	R	S	R	I	S	R	R	R	R	R	R	R	R	S	10
EC91	Kidney	Poultry	2015	S	S	R	I	R	I	S	R	R	R	I	R	R	I	R	S	8
EC92	Mammary gland	Bovine	2018	S	S	I	S	R	I	S	R	S	I	R	R	S	R	R	S	6
EC93	Liver	Poultry	2019	S	S	I	R	R	I	R	R	R	R	R	R	R	R	R	S	11
EC94	Liver	Buffalo	2019	R	S	R	R	R	R	R	R	R	R	R	R	R	R	R	R	15
EC95	Brain	Bovine	2015	R	S	R	I	R	R	S	R	S	R	R	R	R	R	R	S	11
EC96	Liver	Poultry	2015	S	S	R	S	R	I	R	R	S	R	R	S	R	R	R	S	9
EC97	Liver	Bovine	2015	S	S	S	S	R	R	S	R	R	R	R	R	I	R	R	S	9
EC98	Liver	Bovine	2015	S	S	R	S	R	I	S	R	R	R	R	R	R	R	R	S	10
EC99	Liver	Bovine	2015	S	S	R	R	R	I	S	R	R	R	R	R	R	R	R	S	11
EC100	Liver	Poultry	2015	S	S	R	S	R	I	S	R	S	R	R	R	R	R	R	S	9
EC101	Liver	Poultry	2019	R	S	R	R	R	R	R	R	R	R	R	R	R	R	R	R	15
EC102	Liver	Poultry	2019	S	S	I	S	R	R	S	R	R	R	R	R	R	R	R	R	11
EC103	Liver	Poultry	2019	S	S	R	S	R	I	S	S	S	I	I	R	R	R	R	S	6
EC104	Liver	Poultry	2019	S	S	R	S	S	R	S	R	S	R	R	S	R	R	R	S	8

EC105	Liver	Poultry	2019	S	S	R	S	R	R	S	R	S	I	I	R	R	R	R	S	8
EC106	Liver	Poultry	2019	S	S	S	R	S	R	R	R	R	R	R	S	I	R	R	S	9
EC107	Oviduct	Poultry	2019	R	S	R	I	R	R	S	R	R	R	R	R	R	R	R	S	12
EC108	Oviduct	Poultry	2019	R	S	R	I	R	R	R	R	R	R	R	R	R	R	R	S	13
EC109	Joint swab	Poultry	2019	S	S	R	S	R	R	S	R	R	R	R	R	R	R	R	S	11
EC110	Liver	Poultry	2019	S	S	R	S	R	S	S	R	R	R	R	R	R	R	R	S	10
EC111	Joint swab	Poultry	2019	S	S	R	I	R	I	R	R	R	R	R	R	R	R	R	S	11
EC112	Joint swab	Poultry	2019	S	S	R	S	R	I	S	R	I	R	R	R	I	R	R	R	9
EC113	Intestine	Poultry	2019	S	S	R	S	R	I	S	R	S	I	S	R	R	R	R	S	7
EC114	Joint swab	Poultry	2019	S	S	S	S	R	R	S	S	S	I	I	R	S	S	R	I	4

*Disks potencies; R: Resistant; I: Intermediate; S: Sensible; AMI: Aminosidine; COL: Colistin; ENR: Enrofloxacin; LIN/SPE: Lincomycin & Spectomicin; OXY: Oxytetracycline; THM: Thiamphenicol; TLM: Tylmicosin; TYL: Tylosin; THR: Trimethoprim; SUL: Sulphamethoxazole; AMP: Ampicillin; DOX: Doxycycline; FLQ: Flumequine; ERY: Erythromycin; AMX: Amoxicillin; APR: Apramycin.
